# Randomized phase II trial of fulvestrant alone or in combination with bortezomib in hormone receptor-positive metastatic breast cancer resistant to aromatase inhibitors: a New York Cancer Consortium trial

**DOI:** 10.1038/npjbcancer.2016.37

**Published:** 2016-12-14

**Authors:** Kerin Adelson, Bhuvaneswari Ramaswamy, Joseph A Sparano, Paul J Christos, John J Wright, George Raptis, Gang Han, Miguel Villalona-Calero, Cynthia X Ma, Dawn Hershman, Joseph Baar, Paula Klein, Tessa Cigler, G Thomas Budd, Yelena Novik, Antoinette R Tan, Susan Tannenbaum, Anupama Goel, Ellis Levine, Charles L Shapiro, Eleni Andreopoulou, Michael Naughton, Kevin Kalinsky, Sam Waxman, Doris Germain

**Affiliations:** 1 Yale Cancer Center and Smilow Cancer Hospital, Yale University School of Medicine, New Haven, CT, USA; 2 The Ohio State Comprehensive Cancer Center, Ohio State University, Columbus, OH, USA; 3 Department of Oncology, Montefiore Medical Center, Bronx, NY, USA; 4 Department of Healthcare Policy & Research, Weill Cornell Medical Center, New York, NY, USA; 5 Investigational Drug Branch, Cancer Therapy and Evaluation Program, National Cancer Institute, National Institutes of Health, Bethesda, MD, USA; 6 Department of Medicine, Northwell Health, Lake Success NY and Hofstra Northwell School of Medicine, Hempstead, NY, USA; 7 Department of Epidemiology and Biostatistics, School of Public Health, Texas A&M University, College Station, TX, USA; 8 Miami Cancer Institute, Baptist Health South Florida, Miami, FL, USA; 9 Department of Internal Medicine, Washington University School of Medicine, St Louis, MO, USA; 10 Department of Medicine and Epidemiology New York Presbyterian-Columbia University Medical Center, New York, NY, NY, USA; 11 Department of Medicine, Division of Hematology/Oncology, Seidman Cancer Center of the University Hospitals of the Cleveland Medical Center, Cleveland, OH, USA; 12 Tisch Cancer Institute, Icahn School of Medicine at Mount Sinai, Mount Sinai Health System, New York, NY, USA; 13 Division of Hematology and Oncology, Department of Medicine, Weill Cornell Medical Center, New York, NY, USA; 14 Department of Hematology and Medical Oncology, Cleveland Clinic Taussig Cancer Center, Cleveland, OH, USA; 15 Perlmutter Cancer Center, NYU Langone Medical Center, New York University School of Medicine, New York, NY, USA; 16 Department of Medical Oncology, Rutgers Cancer Institute of New Jersey, New Brunswick, NJ, USA; 17 Department of Medicine, University of Connecticut Health Center, Farmington, CT, USA; 18 Roswell Park Cancer Institute, Jacobs School of Medicine and Biomedical Science, State University of New York at Buffalo, Buffalo, NY, USA; 19 Department of Medicine, Division of Hematology and Oncology, New York Presbyterian-Columbia University Medical Center, New York, NY, USA

## Abstract

The proteasome inhibitor bortezomib enhances the effect of the selective estrogen receptor (ER) downregulator (SERD) fulvestrant by causing accumulation of cytoplasmic ER aggregates in preclinical models. The purpose of this trial was to determine whether bortezomib enhanced the effectiveness of fulvestrant. One hundred eighteen postmenopausal women with ER-positive metastatic breast cancer resistant to aromatase inhibitors (AIs) were randomized to fulvestrant alone (Arm A—500 mg intramuscular (i.m.) day −14, 1, 15 in cycle 1, and day 1 of additional cycles) or in combination with bortezomib (Arm B—1.6 mg/m^2^ intravenous (i.v.) on days 1, 8, 15 of each cycle). The study was powered to show an improvement in median progression-free survival (PFS) from 5.4 to 9.0 months and compare PFS rates at 6 and 12 months (*α*=0.10, *β*=0.10). Patients with progression on fulvestrant could cross over to the combination (arm C). Although there was no difference in median PFS (2.7 months in both arms), the hazard ratio for PFS in Arm B versus Arm A (referent) was 0.73 (95% confidence interval (CI)=0.49, 1.09, *P*=0.06, 1-sided log-rank test, significant at the prespecified 1-sided 0.10 *α* level). At 12 months, the PFS proportion in Arm A and Arm B was 13.6% and 28.1% (*P*=0.03, 1-sided *χ*^2^-test; 95% CI for difference (14.5%)=−0.06, 29.1%). Of 27 patients on arm A who crossed over to the combination (arm C), 5 (18%) were progression-free for at least 24 weeks. Bortezomib likely enhances the effectiveness of fulvestrant in AI-resistant, ER-positive metastatic breast cancer by reducing acquired resistance, supporting additional evaluation of proteasome inhibitors in combination with SERDs.

## Introduction

Endocrine therapy prolongs survival in patients with metastatic breast cancer whose tumors express the estrogen receptor (ER) and is better tolerated than chemotherapy. Current options include selective estrogen receptor modulators (e.g., tamoxifen), aromatase inhibitors (e.g., anastrozole, letrozole, and exemestane), and selective estrogen receptor downregulators (SERDs) (e.g., fulvestrant).^1^ However, resistance to endocrine therapy is a major clinical challenge.^2^ Recently, several agents have been shown to enhance the effectiveness of hormonal modulation, including the mTor inhibitor everolimus,^3^ the CDK 4/6 inhibitor palbociclib,^4^ and the histone deacetylase inhibitor entinostat,^5^ indicating that it may be feasible to abrogate endocrine resistance with rational combinations of endocrine and non-endocrine agents, including those that have only modest activity when used alone.

Fulvestrant is a SERD that binds, inhibits, and degrades the estrogen receptor (ER). It binds with 100-fold greater affinity than tamoxifen, and more effectively inhibits estrogen signaling than either tamoxifen or AIs^6–8^ suggesting that it may be a better platform for combining with agents targeting other pathways. In addition to degrading the ER in the nucleus, fulvestrant has a unique mechanism of action, which promotes the accumulation of insoluble ER aggregates in the cytoplasm, activating a sustained unfolded protein response, which leads to DNA fragmentation and apoptosis. Under normal circumstances the proteasome clears these ER aggregates, preventing cell death through this mechanism. By blocking proteasome activity, bortezomib enhances cytoplasmic fulvestrant-mediated ER aggregation, leading to apoptotic cell death in a panel of ER-positive breast cancer cell lines, a tamoxifen-resistant cell line *in vitro*, and also augments tumor regression in a hormone resistant breast cancer xenograft model.^9^ When bortezomib is added to fulvestrant, the nuclear degradation of the ER is maintained. Bortezomib enhances the efficacy of fulvestrant by promoting the novel effect of fulvestrant-mediated ER aggregates in the cytoplasm.

On the basis of these observations, we initiated a hypothesis-driven, randomized phase II open-label trial comparing fulvestrant with fulvestrant plus bortezomib in postmenopausal women with ER-positive metastatic breast cancer with disease that was resistant to AI therapy, a common indication for fulvestrant therapy.^10^ The primary trial endpoint was progression-free survival (PFS), and we herein report the results of the primary analysis. A secondary objective was to determine whether adding bortezomib to fulvestrant produced clinical benefit in patients whose disease progressed on the fulvestrant alone arm.

## Results

### Patient characteristics

One hundred eighteen patients were enrolled from 17 institutions between June 2010 and October 2013, including 59 patients randomized to fulvestrant alone (arm A) and 59 patients randomized to fulvestrant plus bortezomib (Arm B). Two patients randomized to Arm B never received protocol therapy. Of 59 patients randomized to fulvestrant alone, Arm A, 27 (46%) crossed over to receive fulvestrant plus bortezomib (Arm C) at progression on fulvestrant alone.

The baseline characteristics of the 116 treated patients on arm A and B, plus the 27 patients who crossed over from arm A to arm C are shown are shown in Table 1. There were no significant differences in patient characteristics between arms A and B with regard to median age, Eastern Cooperative Oncology Group (ECOG) performance status, prior chemotherapy for metastasis, average prior lines of endocrine therapy (1.43 vs. 1.47), or liver metastases (36 vs. 37%). Patients in arm A had longer median interval between diagnosis and metastasis (49 vs. 28 months) and were more likely to present with *de novo* metastatic disease (32 vs. 26%).

### Efficacy

**Figure 1:**
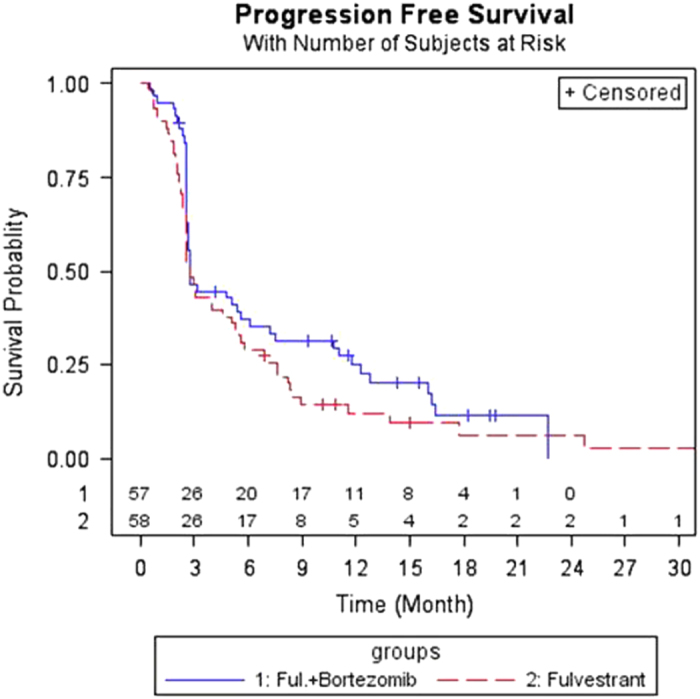
Kaplan–Meier estimates of progression-free survival for patients treated with fulvestrant (arm A) and fulvestrant plus bortezomib (arm B).

PFS rates at 12 months were 13.6% in arm A and 28.1% in arm B (*P*=0.03, 1-sided *χ*^2^-test; 95% confidence interval (CI) for difference (14.5%)=−0.06, 29.1%). PFS rates at 6 months were 28.8% in arm A versus 38.6% in arm B, respectively (*P*=0.13 for one-sided *χ*^2^-test). Although median PFS was similar in the two arms (2.69 vs. 2.73 months, respectively), the hazard ratio for Arm B versus Arm A (referent) was 0.73 (95% CI=0.49, 1.09, *P*=0.06, one-sided log rank test) (Figure 1). This was significant at the prespecified *α* of<0.1.

We also compared the PFS in Arms A and B using the two-time point test procedure as was pre-specified in the protocol.^18^ We implemented the Mantel-Haenszel test procedure proposed by Freidlin and Korn^18^ to adjust for the potential bias between treatment arms due to subjective aspects coming from, for example, as specified in ref. 18, ‘desire of the patient and treating physician to get a patient on an active therapy regimen as quickly as possible.’ If the two time points were chosen to be the median (month 2.71) and twice the median (month 5.42) as suggested in Freidlin *et al.*^18^ the two-sided test *P*-value was not significant (*P*=0.290). However, if we chose the two time points to be months 6 and 12 given that the patient follow-ups were at every 3 months after initiating treatment, the two-sided test *P*-value was significant (*P*=0.059), which was consistent with the significance from the log-rank test.

We used the reduced piecewise exponential estimate^11^ to detect statistically significant changes in the hazard rate (risk) of cancer progression in both arms. Then we compared the two arms using the two-sample exact exponential test developed by Han *et al*^12^ which is the most power unbiased test. Comparing the two arms for three time periods with constant failure rates (before 3 months, between months 3 and 9, and after 9 months), the two-sided exact *P*-value from the two-sample exact exponential test was 0.056, corresponding to the estimated failure rate of 0.167 in Arm A and 0.077 in Arm B in the time period between 3 and 9 months. This analysis indicated that the failure rate in Arm B was 46% of that in Arm A after the first follow-up and before the third follow-up. Thus, the log-rank test, the two time point test, and the exact exponential test all showed that the patients in the bortezomib-containing arm had improved PFS.

**Figure 2:**
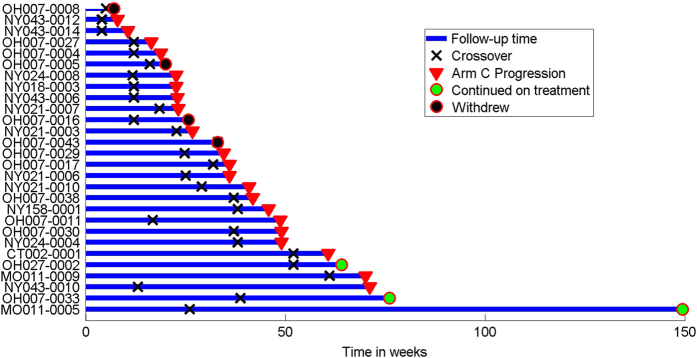
Swimmers plot showing length of time on Arm A and Arm A for patients who crossed over.

Of 27 patients randomized to fulvestrant alone (arm A) who crossed over to fulvestrant plus bortezomib at progression (arm C), clinical benefit occurred in 5 patients (18%) who were progression-free for at least 24 weeks (Figure 2); this is consistent with the 17% CBR rate that was prespecified as potentially promising. In addition, as shown in Figure 2, PFS was substantially longer after crossover to bortezomib than the initial period of PFS with fulvestrant monotherapy in three patients, and three patients remained on combination therapy after crossover at the time of the analysis.

At the time of the PFS analysis, median overall survival had not yet been reached. Death had occurred in 20 of 59 patients (34%) originally randomized to fulvestrant alone, and 15 of 57 patients (26%) originally randomized to and treated with fulvestrant plus bortezomib.

### Treatment administered and adverse events

Patients received a total of 777 cycles of therapy, including 336 cycles on arm A (range 1–33), 333 on arm B (range 1–36), and 108 cycles on arm C (range 1–28). Of the 333 cycles of therapy given in arm B, bortezomib was given at full dose on days 1, 8, and 15 in 294 (88%), 288 (86%), and 282 (95%) of the planned doses, respectively. Bortezomib was discontinued due adverse events in seven patients (12%) in arm B.

Adverse events (all grades) are summarized in Table 2. Adverse events occurred more often in the bortezomib-containing arm, as expected. The most common adverse events in the fulvestrant/bortezomib combination arm compared with the fulvestrant alone arm included nausea (63 vs. 29%), diarrhea (47 vs. 8%), sensory neuropathy (46 vs. 29%), and limb edema (37 vs. 19%). Grade 3 events were uncommon, and there were no grade 4 or 5 adverse events. Peripheral neuropathy observed in both arms at baseline reflected prior taxane therapy and the trial eligibility criteria allowed grade 1 neuropathy. However, post-treatment neuropathy was higher in the bortezomib arm.

## Discussion

We performed a hypothesis-driven, randomized phase II trial of the SERD fulvestrant alone or in combination with the proteasome inhibitor bortezomib in 116 postmenopausal women with ER-positive metastatic breast cancer who had progressive disease after prior aromatase inhibitor therapy. Although previous studies indicated that bortezomib was ineffective when used as monotherapy in patients with metastatic breast cancer,^13,14^ or in combination with aromatase inhibitors,^15^ our trial design was based upon evidence that the fulvestrant-bortezomib combination exhibited synergistic antitumor activity in cell lines *in vitro* and a mouse xenograft model.^9^ The addition of bortezomib to fulvestrant significantly prolonged PFS, the prespecified primary endpoint, resulting in a doubling of the PFS rate at 1 year to 28%, but did not improve median PFS. The overall hazard ratio also favored the combination. The addition of bortezomib to fulvestrant resulted in disease stabilization for at least 24 weeks in 5 of 27 patients (18%) who crossed over to the combination after disease progression on prior fulvestrant monotherapy, providing an additional signal supporting this combination. Adverse events including grade 1–2 nausea, diarrhea, and neuropathy occurred more commonly in the bortezomib-containing arm, but more serious events were uncommon, and only 7 (12%) of patients discontinued bortezomib due to adverse effects, suggesting a favorable therapeutic index for the combination.

At the recommendation of the NCI/CTEP and in published guidelines for randomized phase II clinical trials,^16,17^ we prespecified a statistical design that used a one-sided type I error rate of 10% (*P*<0.1). This is based on the intent to keep patient numbers reasonable in hypothesis generating phase II trials, the goal of which is to identify promising regimens that warrant further study, but not to provide definitive evidence of efficacy.^16^ In addition, our pre-specified statistical design required a test suggested by Friedlin and Korn^17,18^ to eliminate bias that could be introduced by imbalance in visit frequency between arm A (every 4 weeks) and arm B (weekly). Such analysis stipulated that we would look at PFS rates at 6 and 12 months in accordance with timing of imaging studies which were equivalent in both arms. This analysis also demonstrated significance at the prespecified *α* of <0.1. Finally, because our results suggested benefit for the combination by the overall hazard ratio and in PFS rates at 1 year but not in median PFS, we employed a *post hoc* two-sided reduced piecewise exponential approach, with the goal of determining whether clear differences in the fulvestrant only and combination arms emerged within specific time periods. This was also positive with two-sided *P*-value <0.1.

The proteasome normally acts to limit the accumulation of fulvestrant-induced ER aggregates and other toxic cytoplasmic proteins. Our preclinical data demonstrated that the addition of bortezomib to fulvestrant enhanced the accumulation of ER aggregates in the cytoplasm, promoting induction of a proapoptotic unfolded protein response, ultimately leading to cell death.^9^ Fulvestrant's ability to degrade the ER in the nucleus is well described, but its ability to promote aggregation of newly synthesized ER in the cytoplasm has been largely overlooked. As bortezomib does not block the degradation of the ER in the nucleus but enhances the accumulation of ER-aggregates in the cytoplasm, this strategy simultaneously exploits both effects of fulvestrant on the ER. Resistance to endocrine therapy has been categorized as primary, defined as disease progression within 6 months, or secondary, defined as disease progression occurring after 6 months.^3^ Switching to an alternative endocrine therapy^19^ or chemotherapy may be appropriate in patients with either primary or secondary resistance, depending on multiple factors other than the resistance pattern.

Our population exhibited a high degree of primary resistance to fulvestrant, as evidenced by the median PFS in both arms of <3 months, which is similar to that seen in another trial of women treated with fulvestrant after progression on an AI.^20^ Although the median PFS and 6-month PFS rates were similar in the two arms, the 12-month PFS rate was two-fold higher for the combination, suggesting that bortezomib did not impact primary resistance to fulvestrant, but may delay the onset of acquired, or secondary resistance. A potential explanation for this observation is that in breast tumors with primary fulvestrant resistance, the formation of aggregates alone may not be sufficient to induce cell death. Two potential mechanisms support this: First, the level of ER expression is likely to correlate with the ability of bortezomib to mediate its synergistic effect, as previously shown in preclinical models.^21^ As the threshold for the induction of the proapoptotic unfolded protein response requires a minimum level of accumulation of protein aggregates, it is likely that this threshold cannot be reached in breast cancers that express low ER levels. In fact, low ER expression has been shown to correlate with primary resistance to fulvestrant.^22^ Second, elimination of protein aggregates by autophagy provides an alternative mechanism to avoid the induction of the pro-apoptotic unfolded protein response.^23^

Since this trial was initiated, CDK4/6 inhibition has emerged as an important new therapeutic strategy for enhancing the effectiveness of endocrine therapy, with a substantial impact in abrogating primary resistance. The PALOMA 3 trial demonstrated that the combination of the CDK4/6 inhibitor palbociclib with fulvestrant significantly prolonged median PFS compared with fulvestrant alone (median 3.8 vs. 9.2 months, HR 0.42, *P*<0.0001).^24^ Nonetheless, acquired resistance eventually develops in most patients, and other therapeutic strategies are needed to address this clinical problem. Preclinical cell line and xenograft data in multiple myeloma suggest that palbociclib can enhance the cytotoxic effects of bortezomib.^25,26^ A phase I/II study demonstrated that the combination of palbociclib and bortezomib can be safely co-administered.^27^ Given the complementary mechanism of action of palbocilib and bortezomib, their impact on different resistance patterns, and their safety when combined in multiple myeloma, the combination of fulvestrant plus palbocilib and bortezomib may warrant further evaluation in preclinical models and carefully designed clinical trials.

In conclusion, our study supports the hypothesis that adding bortezomib to fulvestrant likely enhances its effectiveness by delaying or reversing acquired fulvestrant resistance. The results of this trial provide a foundation for further exploration of the combination of proteasome inhibitors with SERDs in postmenopausal women with ER-positive metastatic breast cancer as a strategy for addressing acquired resistance to fulvestrant-containing endocrine regimens.

## Materials and methods

### Study design and treatment

This was an open-label, multicenter, randomized phase II trial. All patients received fulvestrant (Faslodex, AstraZeneca Pharmaceuticals LP, Wilmington, DE, USA) at a standard dose and schedule (500 mg IM days −14, 1, 15 in cycle 1, and day 1 of each subsequent cycle). Patients were randomized to Arm A (fulvestrant alone), or Arm B (fulvestrant plus bortezomib (Velcade, Millennium, Boston, MA, USA)). Stratification factors at registration and randomization included ECOG performance status (0 vs. 1–2), measurable disease (yes versus no), and prior chemotherapy for metastatic disease (yes versus no). The bortezomib dose and schedule was 1.6 mg/m^2^ by rapid i.v. infusion over 3–5 seconds on days 1, 8, 15 of each 28-day cycle, a schedule that was shown to be as effective and less toxic in multiple myeloma than the biweekly dosing regimen.^28^ Fulvestrant was initiated two weeks prior to the first dose of bortezomib to ensure adequate fulvestrant blood levels prior to beginning bortezomib.^29^ Patients were allowed to receive up to two doses of fulvestrant administered within a 4-week period and not >6 weeks prior to randomization. Concurrent treatment with bone antiresorptive agents (e.g., bisphosphonates and denosumab) was permitted for patients with bone metastases. Patients with progression on fulvestrant alone (arm A) could cross over at progression to the fulvestrant plus bortezomib combination (arm C). Treatment was continued without interruption until disease progression, severe or intolerable toxicity, or withdrawal of consent. The trial was reviewed, approved, and sponsored by the Cancer Therapy Evaluation Program of the National Cancer Institute (ClinicalTrials.gov, identifier NCT01142401) and coordinated by the New York Cancer Consortium. The local institutional review board at each participating institution approved the protocol. All patients gave written informed consent.

### Eligibility criteria

Postmenopausal women with histologically or cytologically confirmed unresectable locally advanced or metastatic, ER-positive, Her2/neu negative (as defined by local institutional laboratories) breast cancer with measurable and/or non-measurable disease by RECIST 1.1 criteria were eligible. Patients were required to have AI-resistant disease, defined either as relapse while receiving adjuvant AI therapy and/or disease progression after one or more AIs for metastatic disease. Patients were allowed to have prior adjuvant chemotherapy, no more than one prior chemotherapy regimen for metastatic disease and were required to have recovered from prior neuropathy to grade 0–1. Other eligibility criteria included: age ⩾18 years, ECOG performance status of 0 to 2; adequate organ and marrow function (leukocytes ⩾3,000/μl, absolute neutrophil count ⩾1,500/μl, platelet count ⩾100,000/μl, total bilirubin ⩽2.0 mg/dl, AST and/or ALT ⩽2.5× institutional upper limit of normal, serum creatinine ⩽1.5 mg/dl).

### Tumor assessments, clinical evaluations, and dose modifications

All patients underwent computed tomography (CT) of the chest and abdomen and a bone scan within 4 weeks of registration. Tumor response was assessed every 12 weeks (±1 week) after cycle 1, day +1 by CT using RECIST criteria version 1.1,^30^ and bone scans were repeated every 24 weeks (±2 weeks). If the baseline bone scan showed metastases, and if the bones were the only site of non-measurable disease, then bone scan was repeated every 12 weeks (±1 week). Physician visits occurred on day 1 of each cycle for patients in both arms. Adverse events were graded according to the National Cancer Institute Common Terminology for Adverse Events, version 4.0. For patients who experienced febrile neutropenia, grade 4 toxicity hematologic toxicity, grade 2 neuropathy (or grade 1 with pain), or other grade 3–4 non-hematologic toxicity, the bortezomib dose was reduced, first to 1.3 mg/m^2^, then if recurrent to 1 mg/m^2^, and finally to 0.7 mg/m^2^. Fulvestrant was not held if bortezomib was held for toxicity, and no dose reduction of fulvestrant was allowed. Patients who stopped bortezomib due to severe or intolerable toxicity continued fulvestrant alone until disease progression.

### Primary and secondary study end points and statistical plan

The primary end point for the randomized phase II comparison of arm A versus arm B was PFS, defined as time from cycle 1, day 1 of therapy to disease progression or death from any cause. Clinical benefit rate (CBR) was a secondary objective, and was defined as complete or partial response, or stable disease (by RECIST 1.1) for at least 24 weeks. A sample size of 118 (59 in each arm) was prespecified in order to provide sufficient power to detect a 70% improvement in median PFS from 5.4 to 9.0 months, and compare PFS rates after 6 and 12 months (one-sided *α*=0.10, *β*=0.10), with 100 PFS events required to perform the primary analysis. The PFS distributions of the two treatment arms were estimated by Kaplan–Meier survival analysis and compared by an unstratified log-rank test. Ninety-five percent confidence intervals for the Kaplan–Meier PFS estimates were calculated using Greenwood’s formulae. The primary comparison was made using the intent-to-treat (ITT) patient population for all patients who received at least one dose of fulvestrant. Because follow-up visit frequency differed between the two treatment arms, a protocol-specified two time point Mantel–Haenszel test procedure proposed by Freidlin *et al*^18^ was also used to reduce evaluation-time bias that could be introduced when comparing PFS between the two treatment arms.^17^ This technique was applied to test the association of PFS and treatment arm status with the two time points specified at the median and twice the median of the control arm (fulvestrant alone), as well as at months 6 and 12. A reduced piecewise exponential estimate^11^ was also used to fit the survival distribution in the two treatment arms. The two arms were then compared in terms of the piecewise exponential model parameters using a two-sample exact exponential test given in ref. 12.

A secondary objective was to evaluate the CBR in patients who crossed over from arm A (fulvestrant-alone) to arm C (fulvestrant+bortezomib) after progression on fulvestrant alone. The crossover phase was designed to distinguish between a CBR of 5 vs. 25% (*α*=0.10, *β*=0.10) using a Fleming one-stage design. The regimen would be considered worthy of further testing if at least 3 of 18 patients exhibited clinical benefit (i.e., CBR 17%). This assumed that about 30% of patients on arm A would cross over to arm C, although the actual crossover rate (27/59 (46%)) was higher than projected.

## Figures and Tables

**Table 1:** Patient characteristics at registration

	*Arm A: fulvestrant alone*	*Arm B: fulvestrant plus bortezomib*	*Arm C: crossover from A to B*
No. of treated patients	59	57	27
*Age*			
Median	57 years	59 years	57 years
Range	31–83	31–80	31–79
			
*ECOG performance status*			
0	38 (64%)	37 (65%)	19 (70%)
1	20 (34%)	19 (33%)	7 (26%)
2	1 (2%)	1 (2%)	1 (4%)
			
*Measurable disease*			
Yes	38 (64%)	31 (54%)	17 (63%)
No	21 (36%)	26 (46%)	10 (37%)
			
*Metastatic disease sites*			
Bone	45 (76%)	46 (78%)	22 (81%)
Lung	23 (39%)	9 (15%)	9 (33%)
Liver	21 (36%)	22 (37%)	8 (30%)
Pleura	8 (14%)	5 (8%)	2 (76%)
			
*Clinical presentation*			
Metastatic disease at diagnosis	19 (32%)	15 (26%)	17 (63%)
Recurrence after local regional presentation	40 (68%)	42 (74%)	10 (37%)
Interval between diagnosis and metastasis^a^			
Median	49.3 months	29.6 months	47.8 months
Mean	61.0 months	53.9 months	60.9 months
			
*Prior systemic therapy for metastasis*			
Endocrine therapy	39 (66%)	42 (74%)	17 (63%)
Mean no. of prior endocrine therapies for advanced disease	1.43	1.47	
Chemotherapy	16 (27%)	17 (30%)	6 (22%)
Adjuvant chemotherapy	32 (54%)	23 (40%)	13 (48%)
Adjuvant Paclitaxel	18 (30%)	13 (23%)	8 (30%)
Adjuvant Docetaxel	7 (23%)	1 (2%)	2 (7%)
Adjuvant Taxane (sum of paclitaxel and docetaxel)	25 (42%)	14 (25%)	10 (37%)
			
Grade 1 Neuropathy at registration	12 (20%)	12 (21%)	5 (19%)

Abbreviation: ECOG, Eastern Cooperative Oncology Group.

a Includes only patients who presented with localized breast cancer and had recurrence, including 40 patients in arm A, 42 in arm B and 10 patients in arm C.

**Table 2:** Worst grade adverse events by treatment arm

*CTC AE Term*	*Fulvestrant*			*Fulvestrant +Bortezomib*		
	*Grade 1*	*Grade 2*	*Grade 3*	*Grade 1*	*Grade 2*	*Grade 3*
Hematologic	21 (36%)	3 (5%)	0 (%)	27 (47%)	8 (14%)	0 (%)
Anemia	18 (31%)	1 (2%)	0 (%)	18 (32%)	5 (9%)	1 (2%)
Neutropenia	5 (8%)	0 (%)	0 (%)	16 (28%)	1 (2%)	0 (%)
Thromobycopenia	21 (36%)	3 (5%)	0 (%)	27 (47%)	8 (14%)	0 (%)
						
*Metabolic*						
Hyperglycemia	22 (37%)	3 (5%)	1 (2%)	26 (46%)	3 (5%)	0 (%)
Hypoglycemia	2 (3%)	1 (2%)	0 (%)	6 (11%)	1 (2%)	0 (%)
SGOT_AST-High	12 (20%)	3 (5%)	0 (%)	13 (23%)	2 (4%)	2 (4%)
SGPT_ALT-High	6 (10%)	2 (3%)	0 (%)	12 (21%)	0 (%)	0 (%)
Hyponatremia	5 (8%)	0 (%)	0 (%)	6 (11%)	0 (%)	0 (%)
						
*Gastrointestinal*						
Nausea	12 (20%)	4 (7%)	1 (2%)	23 (40%)	11 (19%)	2 (4%)
Vomiting	4 (7%)	3 (5%)	1 (2%)	11 (19%)	6 (11%)	1 (2%)
Diarrhea	4 (7%)	1 (2%)	0 (%)	14 (25%)	8 (14%)	5 (9%)
Constipation	18 (31%)	2 (3%)	0 (%)	19 (33%)	6 (11%)	1 (2%)
Dyspepsia	4 (7%)	2 (3%)	0 (%)	7 (12%)	3 (5%)	0 (%)
Anorexia	3 (5%)	6 (10%)	0 (%)	9 (16%)	4 (7%)	0 (%)
						
*Neurologic*						
Headache	6 (10%)	1 (2%)	0 (%)	11 (19%)	3 (5%)	0 (%)
Pain	18 (31%)	14 (24%)	3 (5%)	16 (28%)	14 (25%)	1 (2%)
Neuropathy (Sensory)	17 (29%)	0 (%)	0 (%)	23 (40%)	3 (5%)	0 (%)
						
*Mucocutaneous*						
Rash/desquamation	5 (8%)	0 (%)	0 (%)	6 (11%)	0 (%)	0 (%)
Injection Site Reaction	13 (22%)	1 (2%)	0 (%)	7 (12%)	0 (%)	0 (%)
						
*Cardiopulmonary*						
Dyspnea	14 (24%)	4 (7%)	1 (2%)	7 (12%)	3 (5%)	1 (2%)
Cough	14 (24%)	2 (3%)	1 (2%)	11 (19%)	2 (4%)	0 (%)
						
*Constitutional and Other*						
Fatigue	30 (51%)	2 (3%)	1 (2%)	20 (35%)	11 (19%)	1 (2%)
Limb edema	9 (15%)	2 (3%)	0 (%)	16 (28%)	5 (9%)	0 (%)
Fever	8 (14%)	1 (2%)	0 (%)	6 (11%)	1 (2%)	0 (%)
Insomnia	13 (22%)	2 (3%)	0 (%)	14 (25%)	5 (9%)	1 (2%)
Hot flashes	20 (34%)	2 (3%)	0 (%)	17 (30%)	1 (2%)	0 (%)
Dizziness	4 (7%)	0 (%)	0 (%)	10 (18%)	1 (2%)	0 (%)
Pruritis	4 (7%)	0 (%)	0 (%)	7 (12%)	2 (4%)	0 (%)
						
*Rheumatologic*						
Arthralgia	15 (25%)	6 (10%)	0 (%)	11 (19%)	5 (9%)	0 (%)
Myalgia	4 (7%)	2 (3%)	0 (%)	6 (11%)	4 (7%)	0 (%)
						
*Other*						
Anxiety	4(7%)	1(2%)	0(%)	8(14%)	3(5%)	0 (%)
Depression	3(2%)	2(3%)	0(%)	6(11%)	2(4%)	0 (%)
Other^a^^,^^b^						

a There was one grade 4 AST elevation in Arm B, which was unrelated to study drug.

b There was one grade 5 cardiac arrest in Arm A, which was unrelated to study drug.
